# Antimicrobial Resistance Gene Patterns in Traditional Montenegrin *Njeguški* Cheese Revealed by qPCR

**DOI:** 10.3390/genes16091089

**Published:** 2025-09-16

**Authors:** Vesna Milanović, Giorgia Rampanti, Andrea Cantarini, Federica Cardinali, Giuseppe Paderni, Aleksandra Martinovic, Andrea Brenciani, Lucia Aquilanti, Andrea Osimani, Cristiana Garofalo

**Affiliations:** 1 Dipartimento di Scienze Agrarie, Alimentari ed Ambientali, Università Politecnica delle Marche, Via Brecce Bianche, 60131 Ancona, Italy; v.milanovic@staff.univpm.it (V.M.); g.rampanti@pm.univpm.it (G.R.); a.cantarini@staff.univpm.it (A.C.); f.cardinali@staff.univpm.it (F.C.); l.aquilanti@staff.univpm.it (L.A.); c.garofalo@staff.univpm.it (C.G.); 2 Centre of Excellence for Digitalisation of Microbial Food Safety Risk Assessment and Quality Parameters for Accurate Food Authenticity Certification, University of Donja Gorica, 81000 Podgorica, Montenegro; giuseppe.paderni@udg.edu.me (G.P.); aleksandra.martinovic@udg.edu.me (A.M.); 3 Dipartimento di Scienze Biomediche e Sanità Pubblica (DSBSP), Università Politecnica delle Marche, Via Tronto 10/A, 60126 Ancona, Italy; a.brenciani@univpm.it

**Keywords:** antimicrobial resistance, carbapenemase encoding genes, dairy products, raw sheep’s cheese, molecular methods, risk assessment

## Abstract

**Background/Objectives**: This study was aimed to investigate the safety profile of traditional Montenegrin *Njeguški* cheese by quantifying genes associated with resistance to clinically important antibiotics. **Methods**: Samples of *Njeguški* cheese were sourced from three artisan producers in Montenegro, identified as A, B, and C, with three individual batches selected per producer. Quantitative PCR (qPCR) was performed on bacterial DNA extracted directly from samples to detect genes encoding resistance to macrolide–lincosamide–streptogramin B (MLS_B_) [*erm*(A), *erm*(B), *erm*(C)], vancomycin (*van*A, *van*B), tetracyclines [*tet*(M), *tet*(O), *tet*(S), *tet*(K), *tet*(W)], β-lactams (*mecA*, *blaZ*), aminoglycosides [*aac (6′)-Ie aph (2″)-Ia*], and carbapenems (*bla*_KPC_, *bla*_OXA-48_, *bla*_NDM-1_, *bla*_GES_, and *bla*_VIM_). **Results**: Among the MLS_B_ resistance genes, *erm*(B) was detected in all samples, *erm*(C) was present only in those from producer B, while *erm*(A) was found exclusively in batch 3 from producer C. Tetracycline resistance genes were widely distributed, except for *tet*(O), which was absent in batch 3 from producers A and B. Regarding β-lactam resistance, both *blaZ* and *mecA* were consistently detected across all samples, with statistically significant differences observed between producers. None of the samples tested positive for vancomycin resistance genes or the aminoglycoside resistance gene, regardless of producer. Among the carbapenemase genes analyzed, *bla*_NDM-1_ was the only one detected, found in most samples from producers B and C. **Conclusions**: This research provides the first risk assessment of artisanal and commercial *Njeguški* cheese regarding antimicrobial resistance genes. The findings offer valuable insights to enhance the microbiological safety of traditional Montenegrin cheeses, supporting consumer confidence in local and international markets.

## 1. Introduction

Over the past few decades, bacteria have increasingly developed resistance to antimicrobials, largely as a result of the selective pressure caused by their wide use and misuse in human medicine, agriculture, aquaculture, and animal farming [1,2,3,4,5,6]. Antimicrobial resistance (AMR) poses a significant threat to public health, with an increasing incidence of treatment failure in infections caused by multidrug-resistant bacterial strains [6,7]. As highlighted by Caniça et al. [5,8], AMR is a complex and dynamic network with resistant bacteria and resistance genes capable of spreading globally across diverse environments, including human, animal, and ecological settings. This is primarily because AMR genes are frequently carried on mobile genetic elements like plasmids and transposons, which facilitate their transfer between bacteria and contribute to the genomic adaptability of bacterial populations. In addition, the transfer of antimicrobial-resistant microorganisms and/or resistance genes to humans through the food chain is an issue of growing concern. Indeed, the food chain is widely acknowledged as one of the primary pathways through which AMR spreads to the human population [1,2,3,4,5,6,7,8,9,10,11,12,13,14,15,16]. Specifically, the consumption of food can introduce bacteria and their AMR genes into the human gastrointestinal tract. Viable bacteria that survive the gastric barrier, as well as free bacterial DNA capable of transforming the human gut microbiota, may contribute to the development of the AMR gene reservoir, thereby facilitating the spread of AMR [1,4]. The emergence of carbapenem-resistant bacteria has further worsened the situation, as carbapenems are regarded as the last line of defense for treating severe infections caused by multidrug-resistant Gram-negative bacteria [5,17].

At the same time, the development and application of rapid, sensitive, and reliable molecular methods for detecting AMR genes in complex food matrices remains a significant challenge [3,9,10]. Among molecular techniques, qPCR provides an effective tool for monitoring the spread of AMR in food products. It also supports the implementation of good manufacturing practices in food production and helps trace spreading to other environments.

Among foods, animal-derived and fermented products are the primary potential vehicles for the diffusion of AMR along the food chain to consumers [1,6,15]. This is due to their typically high bacterial loads, the use of starter cultures, and to their possible cross-contamination with intestines of farm animals [1,15]. Specifically, fermented dairy products have been found strongly linked to the dissemination of AMR genes [18,19,20].

*Njeguški* cheese is a traditional Montenegrin hard, full-fat cheese originally made from raw sheep’s milk. It is well-known and highly appreciated, despite being relatively understudied [21,22,23]. In our previous study by Cardinali et al. [21], artisanal *Njeguški* cheese was thoroughly characterized with respect to its microbiota, physicochemical and morpho-textural properties, biogenic amine content, and volatilome. To complete the characterization of *Njeguški* cheese, the present study aims to assess its safety profile by quantifying genes encoding resistance to six classes of antibiotics commonly used in both animal husbandry and clinical practice, namely tetracyclines, macrolide–lincosamide–streptogramin B (MLS_B_), vancomycin, glycopeptides, β-lactams, and carbapenems (the latter used exclusively in clinical settings). This investigation employs a molecular approach based on qPCR analysis of bacterial DNA directly extracted from cheese samples. Specifically, the target genes namely *tet*(M), *tet*(O), *tet*(K), *tet*(W), *tet*(S), *erm*(A), *erm*(B), *erm*(C), *vanA*, *vanB*, *aac (6′)-Ie aph (2″)-Ia*, *mecA*, and *blaZ* were selected as commonly found in foodborne commensal such as lactic acid bacteria (LAB) as well as in human pathogens [12]. The five carbapenemase encoding genes, *bla*_NDM−1_, *bla*_VIM_, *bla*_GES_, *bla*_OXA−48_, and *bla*_KPC,_ often referred to as the “big five” due to their widespread prevalence, are primarily found within the genomes of *Enterobacteriaceae*, *Pseudomonas* spp., and *Acinetobacter* spp., which are commonly present in fermented foods of animal origin [24]. The previous study on *Njeguški* cheese [21] reported high loads of presumptive lactobacilli and lactococcci, both exceeding 8 log cfu g^−1^, along with relatively high counts of *Enterobacteriaceae* and *Pseudomonas* spp., exceeding 5 log cfu g^−1^. These findings justify the present study, which aims to further highlight safety and health issues related to the production of this cheese.

## 2. Materials and Methods

### 2.1. Samples Collection and Microbial DNA Extraction

Samples collection and microbial DNA extraction were carried out as previously described by Cardinali et al. [21]. In detail, 18 *Njeguški* cheese samples were collected in duplicate from three different production batches (b1, b2, and b3) produced by three independent Montenegrin artisanal cheesemakers (A, B, and C) between May and July 2023. This sampling scheme was designed to capture both inter-producer variability, associated with differences in artisanal practices and milk sources, and intra-producer variability across production batches. Considering the limited production scale of *Njeguški* cheese and the practical constraints of collection, it provided a feasible yet representative framework for characterizing the AMR gene patterns of this traditional product. Each cheese weighed approximately 1 kg, with an average diameter of 14 cm and a height of 3.5 cm. In line with traditional practices, all cheeses were prepared primarily from sheeps’ milk (70%) with a minor inclusion of cow’s milk (30%), using animal rennet and marine salt, and were ripened for approximately 30 days prior to sampling.

After collection, cheeses were sealed in sterile vacuum-packaged polyethylene bags, transported to Italy by express courier under refrigerated conditions in insulated iceboxes, and stored at 4 °C upon arrival until processing. Samples were stored for no longer than 24 h prior to DNA extraction.

For microbial DNA extraction, 10 g of each cheese were aseptically weighed and homogenized in 90 mL of sterile peptone water (1 g L^−1^; Oxoid, Milan, Italy) using a Stomacher 400 Circulator (VWR International PBI, Milan, Italy) at 260 rpm for 5 min. From each homogenate, 1 mL aliquots corresponding to the 10^−1^ dilution were centrifuged at 14,000 rpm for 10 min at room temperature. The supernatants were discarded, and the resulting pellets were subjected to DNA extraction. Total microbial DNA was extracted using the E.Z.N.A. Soil DNA Kit (Omega Bio-Tek, Norcross, GA, USA) according to the manufacturer’s protocol, which incorporates both mechanical disruption and chemical lysis steps to optimize bacterial DNA recovery.

DNA concentrations were determined with the Qubit dsDNA HS Assay Kit (Life Technologies, Milan, Italy), while purity was assessed by spectrophotometric absorbance ratios (A260/A280) using a NanoDrop ND-1000 spectrophotometer (Thermo Fisher Scientific, Wilmington, DE, USA).

To confirm successful extraction of bacterial DNA, 2 µL of each extract were used as template for PCR amplification of the V3–V4 region of the 16S rRNA gene with the primer set and cycling conditions described by Klindworth et al. [25]. Positive and negative controls were included in each PCR run to ensure reliability of amplification, and the products were visualized by electrophoresis on a 1.5% (*w*/*v*) agarose gel.

### 2.2. qPCR Protocols

To quantify genes conferring resistance to clinically relevant antibiotics, qPCR was conducted targeting MLS_B_ resistance genes [*erm*(A), *erm*(B), *erm*(C)], vancomycin resistance genes (*van*A, *van*B), tetracyclines resistance genes [*tet*(M), *tet*(O), *tet*(S), *tet*(K), *tet*(W)], β-lactams resistance genes (*mecA*, *blaZ*), aminoglycosides resistance gene [*aac (6′)-Ie aph (2″)-Ia]*, and carbapenems resistance genes (*bla*_KPC_, *bla*_OXA-48_, *bla*_NDM-1_, *bla*_GES_, and *bla*_VIM_) as previously described by Garofalo et al. [11] and Milanović et al. [26]. Calibration curves were established using serial tenfold dilutions of genomic DNA from 18 reference bacterial strains, each carrying one of the AMR genes under investigation. Assays were run on a CFX Connect Real-Time PCR Instrument (Bio-Rad, Hercules, CA, USA), which computed amplification efficiencies (E) and correlation coefficients (R^2^) based on the slopes of the calibration curves. These curves ranged from below 1 to 7 log gene copies per reaction, and the lowest dilution point with consistent amplification across triplicates was taken as the detection threshold for each gene. Absolute quantification was performed by analyzing sample-derived DNA in parallel with the standard dilutions, with gene copy numbers per reaction calculated from the standard curve slopes. Each qPCR mixture contained: (i) 4 μL of extracted DNA, (ii) 5 μL of Type-it 2X HRM PCR Master Mix (Qiagen, Hilden, Germany); (iii) forward and reverse primers for each AMR gene at the following concentrations: 1000 nM for vancomycin resistance genes, 900 nM for *erm*(B), *tet*(K), *tet*(M), *tet*(O), *tet*(S), and *mecA*, 500 nM for *erm*(A), *blaZ*, and *aac (6′)-Ie aph (2″)-Ia*, 400 nM for *erm*(C) and *tet*(W), and 200 nM for carbapenem resistance genes; and (iv) nuclease-free water, to reach a final reaction volume of 10 μL. Amplification of MLS_B_, tetracycline, β-lactam, aminoglycoside, and vancomycin resistance genes followed a protocol of 5 min at 95 °C, then 40 cycles of 15 s at 95 °C and 30 s at 60 °C. For carbapenem resistance genes, the cycling conditions were 5 min at 95 °C, followed by 35 cycles of 20 s at 95 °C, 45 s at 55 °C, and 30 s at 72 °C. Specificity of amplicons was verified by generating melt curves, incrementally increasing the temperature from 65 °C to 95 °C at 0.2 °C/s. Each run included both no-template controls to exclude contamination and positive controls (genomic DNA from reference strains) to confirm assay performance. Gene copy numbers were determined by comparing sample amplification to the calibration standards, with results expressed as log gene copies per gram of sample. Data were derived from three biological replicates, each run in triplicate, and reported as mean ± standard deviation.

### 2.3. Statistical Analysis

To assess differences among samples, a one-way analysis of variance (ANOVA) was performed, followed by Tukey–Kramer’s Honest Significant Difference (HSD) test to identify specific group differences at a significance threshold of *p* < 0.05. Statistical computations were conducted using JMP software (version 11.0.0, SAS Institute Inc., Cary, NC, USA). To investigate overall trends and associations between AMR gene profiles and samples, Principal Component Analysis (PCA) was conducted using the *factoextra* and *ggplot2* packages in R (version 4.4.3). AMR genes consistently below the detection limit in all samples were excluded from the analysis.

## 3. Results

The concentrations of AMR genes in *Njeguški* cheese samples, expressed as log gene copies per gram of sample, are detailed in Table 1.

Regarding MLS_B_ resistance genes, a wide variability was observed across samples from different producers or batches. The *erm*(A) gene was detected exclusively in b3 samples from producer C (4.50 ± 0.14 log gene copies/g). Samples from producer A showed statistically higher levels of *erm*(B) gene (7.08 ± 0.45 log gene copies/g) compared to producers B and C (6.35 ± 0.23 and 6.20 ± 0.54 log gene copies/g, respectively). Notably, *erm*(C) gene was detected only in samples from producer B, with values ranging between 4.62 ± 0.17 and 5.32 ± 0.10 log gene copies/g.

Tetracycline resistance genes were broadly distributed across samples, except for b3 from producers A and B, in which *tet*(O) was not detected. The *tet*(K), *tet*(M), and *tet*(S) genes were the lowest in samples from producer A, whereas the samples from producer C were characterized by the highest levels of *tet*(W) gene (7.05 ± 0.54 log gene copies/g). The concentration of *tet*(O) gene ranged from 2.49 ± 1.93 to 4.11 ± 0.50 log gene copies/g, with no statistically significant differences among producers.

Regarding the tested β-lactam resistance genes, both *blaZ* and *mecA* genes were consistently detected in all samples. Although intra-producer variation was not statistically significant, inter-producer differences were observed: *blaZ* levels were the highest in samples from producer B, while *mecA* concentrations were highest in samples from producer C.

None of the samples tested positive for vancomycin resistance genes (*vanA* or *vanB*) or aminoglycoside resistance gene *aac (6′)-Ie aph (2″)-Ia*, regardless of the producer.

Among carbapenem resistant genes, *bla*_NDM-1_ was the only gene detected. This gene was present in most samples from producers B and C, showing mean concentrations of 6.07 ± 0.58 and 4.19 ± 3.27 log gene copies/g, respectively.

**Figure 1:**
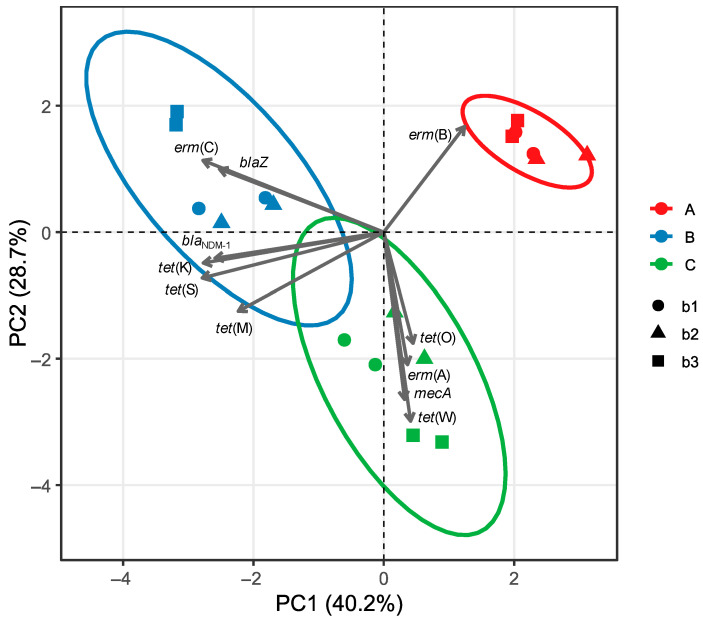
Principal component analysis (PCA) biplot of antimicrobial resistance (AMR) gene profiles in *Njeguški* cheese samples. Ellipses represent 95% confidence intervals for each producer group.

PCA was performed to explore the variation in AMR genes profiles among the *Njeguški* samples. The first two principal components (PC1 and PC2) accounted for 40.2% and 28.7% of the total variance, respectively. The PCA biplot (Figure 1) revealed a clear separation of producers along both axes.

Samples from producer A were distinctly separated from those of producers B and C, primarily along the positive axis of PC1, reflecting elevated levels of *erm*(B) gene. Conversely, samples from producer C clustered toward the lower PC2 region, correlating with higher levels of *tet*(O), *tet*(W), *mecA*, and *erm*(A) genes. Samples from producer B displayed a distinct clustering pattern along the negative axis of PC1, primarily influenced by elevated levels of *erm*(C) and *blaZ* genes. Of note, the close spatial grouping of samples from the same producer and batch suggests a high degree of batch-level consistency in AMR genes composition within each producer.

## 4. Discussion

Very recently, Tiedje et al. [16] highlighted the risks associated with the spread of antimicrobial-resistant bacteria and AMR genes in agri-food production systems, emphasizing the urgent need for rapid and effective measures within the One Health framework. This holistic approach underscores the perspective that the environment, animals, plants, food, feed, and humans are interconnected components of a whole system, interacting with and influencing each other through the exchange of AMR genes and antimicrobial-resistant bacteria. Cheese is one of the most widely consumed foods in the human diet and is often associated with health benefits. It is produced through milk fermentation driven by the metabolic activities of LAB, including genera such as *Lactobacillus*, *Streptococcus*, *Enterococcus*, and *Leuconostoc*. These bacteria can also act as reservoirs of AMR genes, which may be transferred to other bacteria, including pathogenic species [6,20]. Furthermore, factors such as the extensive use and misuse of antibiotics in livestock, inadequate hygiene during food production, improper use of disinfectants, lack of pasteurization, uncontrolled fermentation, food processing conditions involving acidic and osmotic stresses on LAB, as well as prolonged cold storage (around 30 days at 4 °C), all contribute to increasing the frequency of AMR gene transfer [16,20]. Chaves et al. [20] recently reviewed the emergence of AMR among LAB and human pathogens in raw milk cheeses. Specifically, raw sheep’s milk and cheeses, whose global consumption is increasing due to their high nutritional value, can act as a vehicle for the transmission of pathogens and potential pathogens, including AMR strains such as *Staphylococcus aureus*, *Streptococcus* spp., *Enterococcus* spp., *Enterobacteriaceae*, coliforms, *Escherichia coli*, *Pseudomonas aeruginosa*, *Yersinia enterocolitica*, and *Bacillus* spp. [6,27,28,29,30,31,32,33,34,35,36,37,38,39,40]. In the context of public health, the microbiological quality and safety of dairy products are crucial for consumers and depend on various factors, including the health of the animals, seasonal variations, sampling techniques, and adherence to strict hygiene practices both on farms and during processing. For instance, the presence of *S. aureus* is often linked to subclinical mastitis in animals, whereas the detection of *Enterococcus* species, *Enterobacteriaceae*, coliforms, and *E. coli* typically indicates inadequate hygiene measures [27,28]. Meanwhile, Zinno et al. [18] mapped the distribution of antimicrobial-resistant bacteria and AMR genes in animal-based foods, including fermented products, identifying dairy products as the most contaminated food categories, accounting for 56% and 76% of the total foods analyzed, respectively. In this context, effectively monitoring and quantifying genes that confer resistance to antibiotics commonly used in livestock and clinical settings within *Njeguški* cheese—a traditional Montenegrin cheese originally made from raw sheep’s milk that is gaining popularity in both local and global markets—plays an important role in understanding its safety profile.

Specifically, the genes *erm*(A), *erm*(B), and *erm*(C) cause cross resistance to macrolide, lincosamides and streptogramin B (namely MLS_B_ resistance) by encoding erythromycin ribosomal RNA methylases. These enzymes methylate the bacterial ribosome, preventing the binding of MLS antibiotics to their target site [41]. This is the most widespread mechanism of resistance to MLS_B_ in Gram-positive bacteria such as streptococci, *S. aureus* and coagulase-negative staphylococci (CNS). It is primarily associated with the presence of transposons and plasmids that carry the *erm*(A), *erm*(B), and *erm*(C) genes [41]. *Njeguški* cheese microbiota was found to be dominated by *Streptococcus* spp. and *Streptococcus thermophilus*, with high presumptive lactococci counts of about 9 log cfu g^−1^ [21], suggesting a feasible association of these microorganisms with *erm* genes detection. This hypothesis aligns with the findings reviewed by Verraes et al. [15], who reported that *Lactococcus* and *Streptococcus thermophilus* isolates from dairy products frequently exhibit resistance to erythromycin and tetracycline. In the present study, *erm*(B) gene was detected in all *Njeguški* cheese samples, followed by *erm*(C) gene detected in all samples from producer B. The pattern of *erm* genes observed in *Njeguški* cheese samples is consistent with previous studies reporting *erm*(B) gene as the most prevalent, followed by *erm*(C) gene, in dairy products from various countries such as Poland, China, India, Ethiopia, Brazil, Turkey, Spain and Italy [18,20,42,43,44,45].

Tetracycline resistance can occur through various mechanisms, including efflux pumps, protection of ribosomal binding sites, or enzymatic modification of the antibiotic. The *tet*(M), *tet*(O), *tet*(W), and *tet*(S) genes encode ribosomal protection proteins, whereas *tet*(K) gene encodes a tetracycline-specific efflux pump [46,47,48,49]. As expected, *tet* genes were widely distributed among the cheese samples analyzed, likely due to the extensive global use of tetracyclines over the past decades across various ecosystems, including livestock farming, where they have been used to promote animal growth [45,50,51]. As reviewed by Verraes et al. [15], a high frequency of tetracycline and erythromycin resistance has been observed among *Lactobacillus* strains isolated from artisanal cheeses. *Njeguški* cheese is characterized by the dominance of *Lactobacillus* spp. and viable lactobacilli counts reaching up to 8 log cfu g^−1^ [21], suggesting a likely involvement of the cheese’s LAB microbiota in the dissemination of these resistance traits. Overall, *tet*(M), *tet*(O), *tet*(W), *tet*(K), and *tet*(S) have been previously identified as the most prevalent *tet* genes in foodborne LAB using PCR-based assays, supporting the findings of the present study. In particular, *tet*(M) is the most commonly detected gene among *tet* genes in *Lactobacillus* species isolated from dairy products [20,42,45,51].

β-lactam antibiotics, including penicillin and methicillin, are inactivated through the action of the *blaZ* and *mecA* genes. The *blaZ* gene encodes a β-lactamase enzyme that hydrolyzes the β-lactam ring of penicillin, while *mecA* gene encodes penicillin-binding protein 2a (PBP2a), which confers resistance to methicillin [52]. These resistances are mainly related to *S. aureus* strains, the so-called methicillin-resistant *S. aureus* (MRSA) strains that encode both β-lactamase and PBP2a. MRSA strains are frequently resistant to several categories of antibiotics such as macrolides, tetracyclines, and aminoglycosides [52]. CNS isolated from cheeses in Turkey have been found to carry the *mecA* gene [43]. Similarly, *S. aureus* strains isolated from Brazilian raw milk artisanal cheeses were also reported to harbor this gene [53,54,55,56]. By contrast, *S. aureus* strains isolated from 92 samples of raw sheep’s milk cheese from Slovak Republic shown a penicillin-resistant phenotype carrying *blaZ* gene [40]. Similarly, *blaZ* gene was the prevalent β-lactams resistance gene in 35 Brazilian cheeses made from pasteurized bovine milk [51]. Intriguingly, although metataxonomic analysis [21] did not detect staphylococci, the *blaZ* and *mecA* genes were found in all *Njeguški* cheese samples, suggesting that other bacterial groups may be involved in the dissemination of these resistance genes. This hypothesis is supported by the study of Santamarina-García et al. [6], which reports an increasing number of LAB isolates resistant to β-lactams. The ubiquitous presence of *blaZ* and *mecA* genes in the samples analyzed can be attributed to the extensive global use of β-lactams in livestock farming and to the dissemination of AMR genes, which is reflected in the high prevalence of these genes among bacterial isolates from animal-derived products [6,51,56].

The *vanA* and *vanB* genes confer resistance to vancomycin, a glycopeptide antibiotic that inhibits bacterial cell wall synthesis by binding to its precursors. These genes are commonly found in enterococci, staphylococci, and lactobacilli, which act as reservoirs [57]. Enterococci play a crucial role in cheese maturation by contributing to its distinctive aroma, flavor, and texture, and are therefore often used as starter cultures [20,44]. However, *Enterococcus* strains are also known for their capacity to acquire highly transmissible genetic elements such as plasmids, transposons, and insertion sequences. This ability enhances their potential to carry multiple antibiotic resistances, particularly to erythromycin and tetracyclines, and to transfer AMR genes among different bacterial species [20,29,38,44,58]. Specifically, among vancomycin-resistant enterococci (VRE), *Enterococcus faecium* carries the *vanA* gene, which is considered the primary gene responsible for vancomycin resistance. This gene is located on the transposon Tn1546, which is frequently found on plasmids [20,44]. Despite the high viable counts of lactobacilli and lactococci, the predominance of *Lactobacillus* spp., and the presence of *Enterococcus* spp. (albeit at low relative abundances) as revealed by metataxonomic analysis [21], neither *vanA* nor *vanB* genes were detected in the samples analyzed. These findings are consistent with the studies by Chajęcka-Wierzchowska et al. [42] on dairy products from Poland and by Yao et al. [59] on industrial and artisanal cheeses from various regions in China, where no *van* genes were detected using multiplex PCR and qPCR, respectively. Similarly, none of the *Njeguški* cheese samples tested positive for the aminoglycoside resistance gene *aac(6′)-Ie-aph(2″)-Ia*, which is typically located on the Tn5281 transposon in enterococci and confers resistance to gentamicin by producing aminoglycoside-modifying enzymes [60]. Our findings are consistent with the study by Özdemir and Tuncer [61], who reported the absence of the *aac(6′)-Ie-aph(2″)-Ia* gene among 59 high-level aminoglycoside-resistant enterococci isolates from raw milk and dairy products.

Carbapenems are broad-spectrum β-lactam antibiotics commonly used in clinical medicine, but their use is banned in livestock farming worldwide [17,24]. Bacteria develop resistance to carbapenems primarily by acquiring genes that encode carbapenemases that are β-lactamase enzymes capable of hydrolyzing carbapenems and other β-lactam antibiotics by breaking the β-lactam ring [11,17,24,62]. Carbapenem resistance is a relatively recent phenomenon but is rapidly increasing because carbapenemases encoding genes are carried on mobile genetic elements that can be easily transferred horizontally between different bacteria, both commensal and pathogenic. This facilitates the spread of resistance across various reservoirs, ultimately diminishing the effectiveness of carbapenems in treating bacterial infections [11,17,24,62]. Among these genes, *bla*_NDM-1_ was the only one detected in *Njeguški* cheese samples, specifically in those from producers B and C. The *bla*_NDM-1_ gene codify for 17 variants of class B or metallo-β-lactamases (MBLs) called New Delhi metallo-β-lactamase (NDM) that use a zinc ion (Zn^++^) for the hydrolysis of β-lactam ring [17,62]. Typically, this gene has been found in clinically relevant microorganisms as *Enterobacteriaceae* [62]. Therefore, this finding may be associated with the medium to high viable counts of *Enterobacteriaceae* and *E. coli* observed in the *Njeguški* cheese samples, although no significant differences were found among samples from the three producers [21]. The origin of carbapenemase encoding genes in dairy products remains controversial. Al et al. [63] screened 427 raw milk samples for the presence of *bla*_KPC_, *bla*_NDM_ and *bla*_OXA-48_ gene using qPCR and found no evidence of these genes, thereby excluding raw milk as a source for the dissemination of carbapenemase encoding genes. By contrast, as reviewed by Ramírez-Castillo et al. [17], the *bla*_NDM_ gene and its variants have been detected in *E. coli* and *Klebsiella pneumoniae* strains isolated from cattle and milk samples from India, Algeria, Spain, and China. Furthermore, it is well established that livestock animals serve as major reservoirs of multidrug-resistant bacteria, including carbapenem-resistant strains, which can disseminate carbapenem resistance genes to humans either through the food chain or via environments contaminated with animal feces. Specifically, the *bla*_NDM_ gene and its variants have been reported in poultry, swine, dairy and beef cattle livestock, farm environments, raw milk, animals’ feces, seafood, wildlife and companion animals [17]. The detection of *bla*_NDM-1_ in *Njeguški* cheese samples may therefore suggest that potential sources of contamination could originate from the raw milk, farm environment and/or production environment. These findings highlight the importance of surveillance of AMR genes in raw materials, animals from livestock, farm environment, and animal-based products. From a public health perspective, although the incidence of carbapenem resistance genes in the cheeses under study is low, this remains a significant finding due to their clinical relevance, as carbapenems represent the final therapeutic option for managing multidrug-resistant bacterial infections, particularly those causing life-threatening illnesses [11,17,24,62]. Specifically, the presence of *bla*_NDM-1_ in *Njeguški* cheese samples has important implications for consumer health, as NDM-1 enzymes can hydrolyze all β-lactam antibiotics and possess a high potential for rapid dissemination [17,62], thereby facilitating transmission to human microbiota and pathogens, which poses a significant public health risk. Interestingly, according to a recent review by Alvisi et al. [62], international travel and trade have contributed to the global dissemination of NDM-producing bacteria, with evidence of their spread in the Western Balkans.

Overall, this study is consistent with the research by Delannoy et al. [36] on the resistome of 360 raw sheep’s cheeses and 360 raw cow’s cheeses from France, which demonstrated that genes conferring resistance to first-generation β-lactams and tetracyclines are widespread, while those conferring resistance to critically important antibiotics, such as carbapenems, are rare or absent.

Finally, this study revealed a considerable variability in the presence and abundance of AMR genes among samples collected from different artisan producers. The differences in resistance gene profiles suggest that production practices and specific environmental conditions unique to each producer may significantly shape the composition and abundance of AMR genes in the final product. Furthermore, the high consistency in AMR gene profiles within batches from each producer suggests the adoption of stable production practices, highlighting control points for the development of targeted strategies to mitigate AMR. Indeed, the findings of the present study can support the production of safer traditional *Njeguški* cheese, primarily by increasing consumer and producer awareness of the AMR phenomenon along the food chain, and secondarily by guiding the implementation of suitable strategies to monitor and mitigate AMR in these unique artisanal productions. In detail, the primary area of concern lies at the livestock level, where the use of antibiotics must be significantly reduced or entirely avoided to lessen the selective pressure contributing to AMR. At the same time, cheese producers play a crucial role by ensuring careful monitoring of raw materials, ensuring the supply of safe water and maintaining strict hygienic and processing standards throughout cheese production. This includes implementing proper sanitation procedures and waste disposal practices to minimize the environmental release of AMR. To achieve this, workers should receive professional training to adopt effective AMR containment measures across the whole cheese production chain [5,16]. Another effective strategy for mitigating AMR may involve the appropriate screening, selection, and use of autochthonous starter cultures [64], thereby enhancing the quality and safety of cheeses without compromising their traditional character. Intriguingly, very recently the research of Santamarina-García et al. [6] highlighted that cheese production chain, monitored from ovine faeces, ewe milk, whey, fresh cheese, until 60-day-ripened cheese, may have a role in minimizing AMR in the final product since the relative abundance of resistance genes was reduced along the production chain depending on LAB dynamics. In parallel, implementing efficient surveillance systems and conducting research in food production through the use of rapid diagnostic tools that combine culture-based techniques with molecular methods can help identify food-related niches of AMR genes and AMR bacteria. Overall, data collected from food production monitoring systems can guide decision-makers in shaping legislation and policies, as well as in establishing standardized codes of good practice and guidelines designed to support AMR mitigation strategies across all stages of the farm-to-table supply chain [5,16].

## 5. Conclusions

This study represents the first systematic risk assessment of Montenegrin *Njeguški* cheese regarding AMR gene profiles. The high incidence of genes associated with resistance to tetracyclines and β-lactams, combined with the lower and sporadic detection of genes conferring resistance to MLS_B_ and carbapenems, aligns with previously reported data and reflects the long-standing and widespread use of tetracyclines and β-lactams across various ecosystems. While some concerning genes were identified, the overall findings of the present study provide useful guidance for improving microbiological safety and production practices in traditional cheese-making. A limitation of the study could be that it quantifies genes but does not link them to specific bacterial hosts thus limiting interpretation of the potential risk for horizontal gene transfer or pathogenicity. However, the study of AMR genes distribution in cheese samples has been complemented by previous results from viable cell counts and metagenomic sequencing, enabling a feasible correlation between the microbiota of *Njeguški* cheese and the detection of AMR genes. Future research perspectives could include seasonal samplings of the products and longitudinal monitoring to capture variability of AMR genes over time, as well as the selection and application of autochthonous starter cultures not harboring AMR genes. Ultimately, this study underscores the importance of continuous monitoring of fermented foods to evaluate the potential impact of the diet on food safety and public health.

## Figures and Tables

**Table 1:** Log copy number of antimicrobial resistance (AMR) genes detected in *Njeguški* cheese samples.

Producer	Batch	Antimicrobial Resistant Genes (Log Gene Copies/g ± Standard Deviation)																	
		MLS_B_ ^1^			Tetracyclines					β-Lactams		Vancomycin		Aminoglycosides	Carbapenems				
		*erm*(A)	*erm*(B)	*erm*(C)	*tet*(K)	*tet*(M)	*tet*(O)	*tet*(S)	*tet*(W)	*blaZ*	*mecA*	*vanA*	*vanB*	*aac(6′)-Ie-aph(2″)-Ia*	*bla*_OXA-48_	*bla*_VIM_	*bla*_NDM-1_	*bla*_GES_	*bla*_KPC_
A	b1	n.d. ^a^	7.54 ± 0.27 ^a^	n.d. ^a^	4.88 ± 0.19 ^c^	8.26 ± 0.12 ^a^	3.79 ± 0.02 ^a^	9.26 ± 0.06 ^a^	4.83 ± 0.00 ^a^	5.24 ± 0.04 ^a^	3.23 ± 0.30 ^a^	n.d. ^a^	n.d. ^a^	n.d. ^a^	n.d. ^a^	n.d. ^a^	n.d. ^a^	n.d. ^a^	n.d. ^a^
	b2	n.d. ^a^	7.02 ± 0.30 ^a^	n.d. ^a^	5.49 ± 0.06 ^b^	7.72 ± 0.32 ^a^	3.83 ± 0.04 ^a^	8.60 ± 0.28 ^a^	4.86 ± 0.21 ^a^	5.07 ± 0.20 ^a^	3.49 ± 0.07 ^a^	n.d. ^a^	n.d. ^a^	n.d. ^a^	n.d. ^a^	n.d. ^a^	n.d. ^a^	n.d. ^a^	n.d. ^a^
	b3	n.d. ^a^	6.67 ± 0.31 ^a^	n.d. ^a^	6.09 ± 0.01 ^a^	7.61 ± 0.26 ^a^	n.d. ^b^	9.14 ± 0.14 ^a^	4.58 ± 0.08 ^a^	4.96 ± 0.06 ^a^	3.48 ± 0.37 ^a^	n.d. ^a^	n.d. ^a^	n.d. ^a^	n.d. ^a^	n.d. ^a^	n.d. ^a^	n.d. ^a^	n.d. ^a^
	Overall	n.d. ^A^	7.08 ± 0.45 ^A^	n.d. ^B^	5.49 ± 0.55 ^C^	7.86 ± 0.36 ^B^	2.54 ± 1.97 ^A^	9.00 ± 0.35 ^B^	4.76 ± 0.17 ^B^	5.09 ± 0.16 ^B^	3.40 ± 0.25 ^B^	n.d. ^A^	n.d. ^A^	n.d. ^A^	n.d. ^A^	n.d. ^A^	n.d. ^B^	n.d. ^A^	n.d. ^A^
B	b1	n.d. ^a^	6.60 ± 0.16 ^a^	4.84 ± 0.24 ^a^	7.15 ± 0.04 ^a^	8.91 ± 0.30 ^a^	3.74 ± 0.16 ^a^	10.28 ± 0.31 ^a^	4.72 ± 0.11 ^a^	5.42 ± 0.19 ^a^	3.52 ± 0.24 ^a^	n.d. ^a^	n.d. ^a^	n.d. ^a^	n.d. ^a^	n.d. ^a^	6.23 ± 0.25 ^a^	n.d. ^a^	n.d. ^a^
	b2	n.d. ^a^	6.11 ± 0.11 ^b^	4.62 ± 0.17 ^a^	7.23 ± 0.20 ^a^	8.98 ± 0.15 ^a^	3.74 ± 0.01 ^a^	9.91 ± 0.14 ^a^	4.64 ± 0.10 ^a^	5.35 ± 0.15 ^a^	3.40 ± 0.55 ^a^	n.d. ^a^	n.d. ^a^	n.d. ^a^	n.d. ^a^	n.d. ^a^	5.51 ± 0.77 ^a^	n.d. ^a^	n.d. ^a^
	b3	n.d. ^a^	6.35 ± 0.03 ^a,b^	5.32 ± 0.10 ^a^	7.21 ± 0.02 ^a^	8.37 ± 0.01 ^a^	n.d. ^b^	10.65 ± 0.05 ^a^	4.64 ± 0.00 ^a^	5.82 ± 0.05 ^a^	2.97 ± 0.31 ^a^	n.d. ^a^	n.d. ^a^	n.d. ^a^	n.d. ^a^	n.d. ^a^	6.46 ± 0.03 ^a^	n.d. ^a^	n.d. ^a^
	Overall	n.d. ^A^	6.35 ± 0.23 ^B^	4.93 ± 0.35 ^A^	7.20 ± 0.10 ^A^	8.75 ± 0.34 ^A^	2.49 ± 1.93 ^A^	10.28 ± 0.36 ^A^	4.67 ± 0.08 ^B^	5.53 ± 0.25 ^A^	3.30 ± 0.40 ^B^	n.d. ^A^	n.d. ^A^	n.d. ^A^	n.d. ^A^	n.d. ^A^	6.07 ± 0.58 ^A^	n.d. ^A^	n.d. ^A^
C	b1	n.d. ^b^	6.87 ± 0.05 ^a^	n.d. ^a^	7.03 ± 0.24 ^a^	8.75 ± 0.35 ^a^	4.65 ± 0.21 ^a^	9.73 ± 0.05 ^a^	6.70 ± 0.07 ^b^	5.24 ± 0.09 ^a^	5.65 ± 0.67 ^a^	n.d. ^a^	n.d. ^a^	n.d. ^a^	n.d. ^a^	n.d. ^a^	6.49 ± 0.39 ^a^	n.d. ^a^	n.d. ^a^
	b2	n.d. ^b^	5.88 ± 0.28 ^b^	n.d. ^a^	6.17 ± 0.27 ^a^	8.16 ± 0.15 ^a^	4.03 ± 0.39 ^a^	9.74 ± 0.22 ^a^	6.70 ± 0.00 ^b^	5.04 ± 0.21 ^a^	4.76 ± 0.58 ^a^	n.d. ^a^	n.d. ^a^	n.d. ^a^	n.d. ^a^	n.d. ^a^	6.07 ± 0.61 ^a^	n.d. ^a^	n.d. ^a^
	b3	4.50 ± 0.14 ^a^	5.85 ± 0.06 ^b^	n.d. ^a^	6.24 ± 0.22 ^a^	8.63 ± 0.09 ^a^	3.64 ± 0.06 ^a^	10.09 ± 0.14 ^a^	7.75 ± 0.10 ^a^	5.10 ± 0.04 ^a^	4.65 ± 0.20 ^a^	n.d. ^a^	n.d. ^a^	n.d. ^a^	n.d. ^a^	n.d. ^a^	n.d. ^b^	n.d. ^a^	n.d. ^a^
	Overall	1.50 ± 2.32 ^A^	6.20 ± 0.54 ^B^	n.d. ^B^	6.48 ± 0.46 ^B^	8.51 ± 0.33 ^A^	4.11 ± 0.50 ^A^	9.85 ± 0.22 ^A^	7.05 ± 0.54 ^A^	5.13 ± 0.14 ^B^	5.02 ± 0.63 ^A^	n.d. ^A^	n.d. ^A^	n.d. ^A^	n.d. ^A^	n.d. ^A^	4.19 ± 3.27 ^A^	n.d. ^A^	n.d. ^A^

^1^ MLS_B_, macrolide–lincosamide–streptogramin B; n.d., not detected (detection limit for all tested AMR genes: < 1 log gene copy per reaction). Within each column, means followed by different lowercase letters indicate significant differences (*p* < 0.05) among different batches, and capital letters indicate significant differences (*p* < 0.05) among different producers.

## Data Availability

The original contributions presented in this study are included in the article. Further inquiries can be directed to the corresponding author.

## Abbreviations

The following abbreviations are used in this manuscript:

AMR	Antimicrobial Resistance
CNS	Coagulase-Negative Staphylococci
LAB	Lactic Acid Bacteria
MLS_B_	Macrolide–Lincosamide–Streptogramin B
MRSA	Methicillin-Resistant *Staphylococcus aureus*
PBP2a	Penicillin-Binding Protein 2a
PCA	Principal Component Analysis
PCR	Polymerase Chain Reaction
qPCR	Quantitative Polymerase Chain Reaction
VRE	Vancomycin-Resistant Enterococci
